# Editorial: Dietary Lipid Oxidation and Fried Food Toxicology

**DOI:** 10.3389/fnut.2022.858063

**Published:** 2022-03-04

**Authors:** Martin Grootveld, Paul B. Addis, Adam Le Gresley

**Affiliations:** ^1^Leicester School of Pharmacy, De Montfort University, Leicester, United Kingdom; ^2^Department of Food Science and Nutrition, Saint Paul, MN, United States; ^3^School of Life Sciences, Pharmacy and Chemistry, Kingston University, Kingston upon Thames, United Kingdom

**Keywords:** lipid oxidation, frying and quality assurance tests, aldehydes, cardiovascular diseases, inflammation, frying oil

Mankind is facing some difficult challenges and decisions. Indeed, our recently evidenced vulnerability to emergent viruses and how they can best be defended against; whether or not it is wise to totally move to a “carbon-free” world economy; and should we switch from animal foods to plant-based “fake meats,” to name but a few. The human condition, as affected by diet, has been debated for more than a century. It is therefore crucial that decisions made on future human health prospects, and the global environment, are based on sound scientific principles, and not simply to impart an economic advantage of one particular industry or corporation over another.

The historic view that saturated fat and cholesterol are linked to cardiovascular disease (CVD), has experienced a significant evolution. Cholesterol is now considered ***not*** to represent a serious dietary issue, according to the American Heart Association (1). Likewise, the extreme resistance of saturated fat to lipid oxidation, and the lack of hypercholesterolemic effects of some saturated fatty acids, has led to a less severe attitude being expressed by many researchers (1). These new conclusions raise the question of what dietary components are related to human CVD and other pathologies?

One clearly valuable answer to this question is afforded by groups of publications focused on fried food toxicology. It is interesting and perhaps significant that a recent review has summarized intelligibly, the massive amount of evidence adversely implicating omega-6 rich vegetable oils in the development, progression and pathogenesis of human CVDs (2), citing the fact that the introduction of soybean oil in the USA coincides clearly with an increased incidence of this non-communicable chronic disease (NCD). Animal fat consumption has been declining for more than a century, beginning at the same time period that vegetable oil intake began to significantly increase. Interestingly, the authors of Ref. (2) cite the oxidized linoleic acid hypothesis, naming compounds which they specify as causative agents, particularly linoleoylglycerol oxidation products. This hypothesis coincides with the primary focus of the following papers-namely, radical-induced lipid oxidation is a serious health issue for the world's human population and this subject has been “flying under the radar” for far too long.

The first contribution toward this novel series is entitled **“*Evidence-based*** ***challenges to the continued recommendation and use of peroxidatively-susceptible*** ***polyunsaturated fatty acid-rich culinary oils for high-temperature frying practices:*** ***Experimental revelations focused on toxic aldehydic lipid oxidation products”*** by Grootveld. In this study, the toxicities and deleterious health effects of dietary lipid oxidation products (LOPs) are critically evaluated to present an outright challenge to health, food and nutrition researchers who remain as supporters of the postulate that polyunsaturated fatty acid (PUFA)-rich vegetable-based frying oils are “safe,” and without any adverse effects, when used for high-temperature frying episodes. The production, physiological fates and potential deleterious health effects of less commonly considered LOPs, e.g., epoxy-fatty acids, are also delineated. Along with this information, data available on the dietary intake of fried foods by humans are critically examined, with a major focus on the consumption of French fry products. Although a systematic review and update on estimates of quantities of French fry food-sourced dietary LOPs is also provided (specifically, levels of total (*E*)-2-alkenals, (*E,E*)-alka-2,4-dienals, 4-hydroxy-(*E*)-2-alkenals and *n*-alkanals), it should be noted that currently, governmental health authority values for tolerable daily intakes (TDIs) of these toxins are unfortunately limited to acrolein and other lower aldehydic homologs, notably acetaldehyde and formaldehyde. The volatilities of aldehydes is also considered, this being of considerable importance concerning possible toxic effects mediated by their inhalation during the fast-food frying or wok cooking of many foods by restaurant staff, or even domestically. These fumes most especially are generated from the exposure of unsaturated fatty acid (UFA)-rich cooking oils to temperatures ranging from 160°C to sometimes >250°C.

This Commentary paper also reviews the biochemical reactivities of different classes of aldehydes, and their metabolism and toxicological effects, together with problems encountered with the Cramer classification of these agents (3). Of particular importance, the mutagenicities, genotoxicities and carcinogenic properties of aldehydes are evaluated, and subsequently the blood plasma concentrations of these toxins, and their probable dietary and/or endogenous sources, are reviewed. A novel canonical correlation analysis is also performed, in order to explore relationships between the frying oil and corresponding fried potato chip contents of the predominant aldehydic LOPs found in these samples [(*E*)-2-alkenals, (*E,E*)-alka-2,4-dienals and *n*-alkanals)] as a function of consecutive frying periods. Additional attention is also drawn to the supplementation of foods provided to production animals such as chickens and swine with already oxidized (used) frying oils, this apparently representing an important “energy source” for such livestock. This section is largely focused on the deleterious effects exerted by oil-derived LOPs on the growth performance of such production animals. Also explored are delivery methods and administration routes utilized for evaluating the adverse health effects of aldehydic LOPs in animal model systems. This work emphasizes critical requirements for the institution of TDI values for a much broader spectrum of aldehydic LOPs, and also the establishment and performance of future nutritional and epidemiological trials to determine correlations between the frequencies and levels of their dietary consumption, and the incidence and severity of NCDs.

In the contribution by Leong (“***Effect of lipid*** ***oxidation products on inflammation-mediated hypertension and atherosclerosis: A*** ***mini review***”), **t**he regular dietary intake of foods fried or cooked using reheated or thermally-stressed PUFA-rich culinary oils may serve to dysregulate antioxidant activities *in vivo*, a process leading to uncontrolled and hence excessive levels of oxidative stress and inflammation. Aldehydic LOPs act as important agents in the field of inflammation, which is broadly linked to atherosclerosis and its cardiovascular disease sequelae, together with hypertension. To date, much evidence has revealed the adverse effects of such aldehydes through a range of mechanisms. The review presented by Xin-Fang Leong provides a valuable update on the deleterious health effects of aldehydic LOPs through inflammatory signaling pathways, which may act as potential therapeutic targets available for the mediation of cardiovascular diseases. Both malondialdehyde and 4-hydroxy-*trans*-2-alkenals served as critical model aldehydic LOPs for these studies.

In the paper presented by Adam Le Gresley et al. (“***Real-world evaluation of lipid oxidation products and chemical elements in French fries*** ***from two chain fast-food restaurants***”), the concentrations of aldehydes and further LOPs, and trace metal ions, present in French fry and corresponding frying oil samples acquired from two global chain fast-food restaurants, were investigated. LOPs were determined by high-resolution ^1^H nuclear magnetic resonance (NMR) analysis, whilst metal ion levels were evaluated using inductively-coupled plasma optical emission spectrometry (ICP-OES) analyses This work is appropriately described as a “real-world” evaluation, and it represents one of the very first of its kind; indeed, samples from two different well-known global fast-food chain restaurants were featured Moreover, to date little or no investigations of this type were found by the authors, and this study highlighted differing regimens employed by restaurants to diminish LOP concentrations in cooking oils between the two different restaurants involved. Especially interesting are studies performed on the diurnal dependence of these concentrations, and the investigators found that there were indeed significant differences “between-daily time-points”, some of which may arguably arise from replenishments of culinary oils between frying cycles. However, in general it was found that the replenishment of culinary frying oils did not alleviate LOP levels for any significant time period, and therefore the direct prevention of LOP formation by alternative approaches appears to offer some benefits and improvements. However, total French fry lipid acylglycerol contents were consistently higher in samples provided by one restaurant than the other, as indeed were those of the aldehydic LOPs (*E*)-2-alkenals and (*E,E*)-2,4-alkadienals. Moreover, there were also significant “between-restaurant” differences in the French fry oils' iron and aluminum concentrations; notwithstanding, higher levels of redox-active metal ions were not found to be associated with higher concentrations of LOPs found therein.

A further paper by this group, entitled “***The role of polydimethylsiloxane in suppressing the*** ***evolution of lipid oxidation products in thermo-oxidized sunflower oil: Influence of stirring*** ***processes***” (Ampen et al.), explores the abilities of increasing added concentrations of polydimethylsiloxane (PDMS), a valuable anti-foaming agent, to block the O_2_-mediated, free radical-promoted peroxidation of PUFAs in sunflower oil during its exposure to standard frying practices (specifically, continuous thermal-stressing episodes at 180°C for a duration of 300 min.), either unstirred or stirred. No oil replenishment was featured. The results from these investigations clearly demonstrated that the time-dependent extent of suppression of UFA peroxidation observed was highly correlated with increasing PDMS concentration, with mean, added concentration-dependent inhibitions of 15–45% observed for unstirred samples containing up to 0.05 ppm of this agent. The minimum added “concentration” of PDMS required to significantly block LOP formation in both stirred and unstirred culinary oils was found to be only 6.25 ×10^−7^ ppm. Therefore, it certainly appears that only very low levels of added PDMS, which forms an O_2_-inpentratable layer on the surface of culinary frying oils, are sufficient to provide a perhaps acceptable LOP production-dampening effect in PUFA-rich sunflower oils during their exposure to high-temperature frying episodes. The authors also suggest that through appropriate synthetic chemical routes, the PDMS base molecule could be structurally-modified to include an antioxidant functional group such as a phenolic moiety; application of such a novel agent should, at least in principle, enhance these already excellent LOP-suppressive actions.

Finally, a contribution by Kuhnert and Bikaki, entitled “***Thermal peroxidation” of dietary pentapeptides yields*** ***N-terminal*** ***1,2-dicarbonyls***' provides evidence that peptides thermo-oxidatively deteriorate through a mechanism analogous to that of the lipid peroxidation process, and generates a range of novel degradation products of possible toxicological importance. Indeed, at temperatures above the standard frying temperature (180°C), pentapeptides containing an N-terminal phenylalanine residue react through a debenzylation process to produce 1,2-dicabonyl compounds. For this reaction, a radical-based mechanism was proposed, i.e., one which gives rise to a common peroxo-aminal intermediate for two distinct classes of products containing terminal α-1,2-diamide or α-amide-aldehyde functions. For these pentapeptide “peroxidation” processes, the reaction products and mechanisms involved are somewhat aberrant, and have a high level of novelty. They also provide some new perceptions regarding chemical processes employed in food processing. Therefore, this study paves the way for studies focused on the identification and quantification of these novel peroxidised peptide adducts in fried foods, along with other food products available for human consumption. Hence, UFAs are clearly not alone in being able to participate in peroxidation reactions and their associated degradative routes.

## Author Contributions

All authors provided a strong contribution toward this work, which was directed by MG.

## Conflict of Interest

The authors declare that the research was conducted in the absence of any commercial or financial relationships that could be construed as a potential conflict of interest.

## Publisher's Note

All claims expressed in this article are solely those of the authors and do not necessarily represent those of their affiliated organizations, or those of the publisher, the editors and the reviewers. Any product that may be evaluated in this article, or claim that may be made by its manufacturer, is not guaranteed or endorsed by the publisher.
